# Fibrinogen γ′ promotes host survival during *Staphylococcus aureus* septicemia in mice

**DOI:** 10.1016/j.jtha.2023.03.019

**Published:** 2023-03-30

**Authors:** Oscar Negrón, Miranda Weggeman, Jos Grimbergen, Emily G. Clark, Sara Abrahams, Woosuk S. Hur, Jaap Koopman, Matthew J. Flick

**Affiliations:** 1 Department of Pathology and Laboratory Medicine, University of North Carolina at Chapel Hill, Chapel Hill, North Carolina, USA; 2 Lineberger Comprehensive Cancer Center, University of North Carolina at Chapel Hill, Chapel Hill, North Carolina, USA; 3 UNC Blood Research Center, University of North Carolina at Chapel Hill, Chapel Hill, North Carolina, USA; 4 Fibriant BV, Leiden, The Netherlands

**Keywords:** bacteremia, fibrinogen, *S. aureus*, sepsis

## Abstract

**Background:**

*Staphylococcus aureus* is a common gram-positive bacterium that is the causative agent for several human diseases, including sepsis. A key virulence mechanism is pathogen binding to host fibrinogen through the C-terminal region of the γ-chain. Previous work demonstrated that *Fgg*^*Δ5*^ mice expressing mutant fibrinogen γ^Δ5^ lacking a *S. aureus* binding motif had significantly improved survival following *S. aureus* septicemia. Fibrinogen γ′ is a human splice variant that represents about 10% to 15% of the total fibrinogen in plasma and circulates as a fibrinogen γ′-γ heterodimer (phFibγ′-γ). The fibrinogen γ′-chain is also expected to lack *S. aureus* binding function.

**Objective:**

Determine if human fibrinogen γ′-γ confers host protection during *S. aureus* septicemia.

**Methods:**

Analyses of survival and the host response following *S. aureus* septicemia challenge in *Fgg*^*Δ5*^ mice and mice reconstituted with purified phFibγ′-γ or phFibγ-γ.

**Results:**

Reconstitution of fibrinogen-deficient or wildtype mice with purified phFibγ′-γ prior to infection provided a significant prolongation in host survival relative to mice reconstituted with purified phFibγ-γ, which was superior to that observed with heterozygous *Fgg*^*Δ5*^ mice. Improved survival could not be accounted for by quantitative differences in fibrinogen-dependent adhesion or clumping, but phFibγ′-γ-containing mixtures generated notably smaller bacterial aggregates. Importantly, administration of phFibγ′-γ after infection also provided a therapeutic benefit by prolonging host survival relative to administration of phFibγ-γ.

**Conclusion:**

These findings provide the proof-of-concept that changing the ratio of naturally occurring fibrinogen variants in blood could offer significant therapeutic potential against bacterial infection and potentially other diseases.

## 1 | INTRODUCTION

*Staphylococcus aureus* is a gram-positive pathogen and causative agent for illnesses ranging from minor skin infections to serious and potentially life-threatening conditions [1–3]. These infections are particularly problematic in hospital settings where individuals are often immunocompromised. Indeed, prominent vehicles for infection by *S. aureus* are foreign bodies such as catheters, surgical implants, and sutures [4,5]. The transition from a local to a systemic infection occurs once *S. aureus* enter the blood (ie, bacteremia), which can result in sepsis, a major life-threatening disease [6]. The emergence of antibiotic-resistant strains of *S. aureus* (eg, methicillin- and vancomycin-resistant *S. aureus*) are particularly problematic for treatment and has driven the need for novel strategies distinct from classic antibiotic approaches.

Fibrinogen is a major soluble plasma glycoprotein and a dimeric molecule, consisting of 2 pairs of 3 polypeptide chains designated Aα, Bβ, and γ that are connected by disulfide bridges. Beyond playing a key role in clot formation and controlling hemorrhage, fibrinogen can serve as an early line of host defense by limiting pathogen growth and mediating antimicrobial mechanisms against pathogens. However, *S. aureus* has evolved to counteract fibrinogen-mediated antimicrobial function by producing several virulence factors that engage and activate host clotting proteins [7–11]. Fibrinogen-binding proteins are a particularly prominent class of *S. aureus* virulence factors [12–14]. *S. aureus* fibrinogen binding proteins engage multiple domains on fibrinogen, but the C-terminal portion of the fibrinogen γ-chain is a core binding motif for several important *S. aureus* virulence factors. For example, clumping factor A (ClfA) is a cell wall anchored protein that binds the C-terminal portion of the γ-chains and promotes fibrinogen-mediated bacterial clumping in suspension and bacterial adhesion to fibrinogen-coated surfaces [10,13,15]. ClfA further promotes pathogen virulence in sepsis by inhibiting complement activation and neutrophil and macrophage phagocytosis [10,16,17]. Previous work demonstrated that *Fgg*^*Δ5*^ mice carrying a mutant form of fibrinogen lacking the final 5 amino acids of the fibrinogen γ-chain, residues essential for ClfA binding, had significantly prolonged survival compared to wildtype (WT) mice following an *S. aureus* bacteremia challenge and that purified fibrinogen γ^Δ5^ failed to support *S. aureus* clumping and adhesion [18].

Circulating fibrinogen is a heterogeneous mixture of several variants that occur in the blood of all healthy individuals and are the result of an alteration in either the Aα chains or γ chains. The major form of human fibrinogen in circulation consists of Aα chains that are 610 amino acids in length, Bβ chains of 461 amino acids and γ chains of 411 amino acids. Fibrinogen γ′ is a naturally occurring fibrinogen variant that circulates at concentrations ranging from 8% to 15% of total fibrinogen in plasma [19]. It is the product of alternative splicing of *FGG* mRNA and results in the substitution of the final 4 amino acids of the fibrinogen γ-chain with a 20 amino acid sequence [20,21]. Fibrinogen γ′ shares some structural and functional similarities with fibrinogen γ^Δ5^, namely the loss of key residues required for ClfA binding [10,15]. Here, we investigated the hypothesis that fibrinogen γ′ would confer host protection in a manner similar to that observed with *Fgg*^*Δ5*^ mice following a *S. aureus* bloodstream infection.

## 2 | MATERIAL AND METHODS

### 2.1 | Bacteria and growth conditions

WT and ClfA- USA300 as well as WT green fluorescent protein (GFP)-expressing Newman *S. aureus* were kindly provided by Magnus Höök [22]. Stationary phase bacteria were grown in tryptic soy broth (Difco Laboratories) at 37°C overnight, washed, and resuspended in phosphate-buffered solution (PBS) and diluted to an optical density (OD) at 600 nm of 0.4, 1.0, or 6.0, based on the assay. The precise number of bacteria for each assay were determined by a colony forming units (CFUs) assay.

### 2.2 | Fibrinogen purification and recombinant fibrinogen production

Mouse fibrinogen was purified from citrate plasma isolated from naïve WT, heterozygous, and homozygous *Fgg*^*Δ5*^ mice by ammonium sulfate or glycine precipitation, as previously described [23]. In each case, the final fibrinogen pellet was resuspended in HEPES-buffered saline (20 mM HEPES pH 7.4, 150 mM NaCl, and 5 mM ε-amino-n-caproic acid). Notably, fibrinogen purified using each of the methods performed identically in the *in vitro* bacterial adhesion and clumping assays. Human plasma fibrinogen variants (phFibγ-γ and phFibγ′-γ) were purified from commercially available plasma fibrinogen (FIB3, Enzyme Research Laboratories) by anion exchange chromatography using a trimethylaminoethyl resin. The resulting subfractions were concentrated and diafiltered to formulation buffer (either PBS or 5 mM citrate, 50 mM L-arginine with 100 mM NaCl at pH 7.3) by tangential flow filtration using a 100 kDa cut-off filter and sterile filtered. Purity was estimated at >99% by Sodium dodecyl-sulfate polyacrylamide gel electrophoresis (SDS-PAGE) and western blot analysis using a sheep anti-human fibrinogen γ′ antibody (Santa Cruz).

Recombinant human fibrinogen variants (rec-hFibγ-γ and rechFibγ′-γ′) were produced by Chinese hamster ovary cells in stirred tank reactors, using fed batch cell culture and serum-free cell culture media. Following a 10-day production time, cell culture harvest was clarified by depth filtration, and the recombinant fibrinogen protein was purified using a custom-made affinity resin (Life Technologies Corporation) based on the Gly-Pro-Arg-Pro peptide, additional chromatography and filtration steps (details not disclosed for proprietary reasons). The final formulation was performed identical to the formulation of the plasma-derived fibrinogen variants as described above. Purity was >99% as assessed by SDS-PAGE analysis and host cell protein enzyme-linked immunosorbent assay (ELISA) (CHO HCP ELISA Kit, 3G, F550–1 Cygnus).

### 2.3 | Fibrinogen–*S. aureus* adhesion

Bacterial adhesion was analyzed in 96-well NUNC plates (Thermo Fisher) coated with dilution buffer (15 mM Na_2_HCO_3_, 35 mM NaHCO_3_, 3.2 mM NaN_3_) and incubated overnight at 4°C. Plates were then washed 3 times with wash buffer (150 mM NaCl and 0.01% Tween-20), blocked with 1% BSA, 0.05% Tween-20 solution in PBS for 1 hour at 37°C, and subsequently washed 3 times with wash buffer. *S. aureus* was added and incubated for 2 hours at 37°C. Plates were washed 3 times, fixed for 30 minutes with 25% formaldehyde solution, washed, and stained with 0.1% crystal violet. Bound crystal violet was solubilized in 10% acetic acid and quantified in a plate reader at 570 nm.

### 2.4 | Fibrinogen–*S. aureus* clumping

Bacterial clumping in solution was analyzed with purified mouse or human fibrinogen diluted in PBS at concentrations ranging from 0.25 to 25 μg/mL. A suspension of stationary phase cultures of WT or ClfA-*S. aureus* USA300 with an OD at 600 nm of 6.0 were added to 96-well tissue culture plates containing fibrinogen solutions. The plates were agitated using an orbital shaker for 5 minutes followed by measurement at 570 nm to quantify the level of bacterial clumping. For imaging studies, stationary phase WT USA300 or Newman GFP-expressing *S. aureus* were similarly prepared and analyzed in 48-well plates. After the 5 minute clumping reaction, images were captured by either standard brightfield or fluorescent microscopy.

### 2.5 | Bacteremia infection model and treatment of mice with purified fibrinogen

Animal studies were approved by the Institutional Animal Care and Use Committees of Cincinnati Children’s Hospital Medical Center or the University of North Carolina at Chapel Hill. Age matched (>8 weeks) male and female mice on a C57Bl/6J background were used. Human fibrinogen levels in mice were determined following retroorbital (RO) injection of 2 mg of purified human fibrinogen into C57Bl/6 mice. Blood was collected from the inferior vena cava into citrate at defined time points. Platelet poor plasma was analyzed by ELISA using a monoclonal anti-human fibrinogen (Y-18) described previously [24] as capture antibody and a goat anti-human fibrinogen conjugated to peroxidase as detecting antibody. Fibrinogen-deficient (Fga^−/−^) and *Fgg*^*Δ5*^ mice have been previously described [25,26]. Mice were injected with *S. aureus* USA300 and 6 mg of purified fibrinogen via RO injection in the orbital plexus opposite that for which *S. aureus* was delivered. In studies of prophylactic fibrinogen treatment, *Fga*^−/−^ mice were injected 24 hours prior to *S. aureus* challenge to ensure these animals tolerated the bolus of fibrinogen. WT mice were injected 30 minutes prior to *S. aureus* challenge. For analyses of bacterial burden and host responses, *Fga*^−/−^ mice were similarly treated with 6 mg of fibrinogen or vehicle control 24 hours prior to RO infection with *S. aureus* USA300, and mice were euthanized 8 hours after infection for collection of plasma and organs. Complete blood count analyses on whole blood were performed with an Element HT5 (Heska). Bacterial burden was determined for whole blood or tissue homogenates by serial dilution and CFU analysis. Cardiac troponin I (cTnI) levels in plasma were determined by ELISA using a high-density mouse cTnI kit (Life Diagnostics, Inc). Plasma alanine aminotransferase levels were determined using an enzyme assay kit (Labs Biotechnology). In studies where fibrinogen was given as a therapeutic treatment, mice were injected 30 minutes after *S. aureus* challenge. In all survival studies, moribundity defined as a state of nonrecovery was used as a humane endpoint.

### 2.6 | Statistical analyses

All analyses were performed using Prism 9. Comparisons of multiple groups were performed using analysis of variance (anova) and Tukey’s multiple comparison test. Analyses of survival were performed using the Kaplan-Meier method. Results were considered significant when *p* < .05.

## 3 | RESULTS

### 3.1 | Host protection from S. aureus septicemia in mice expressing fibrinogen γ^Δ5^

**FIGURE 1:**
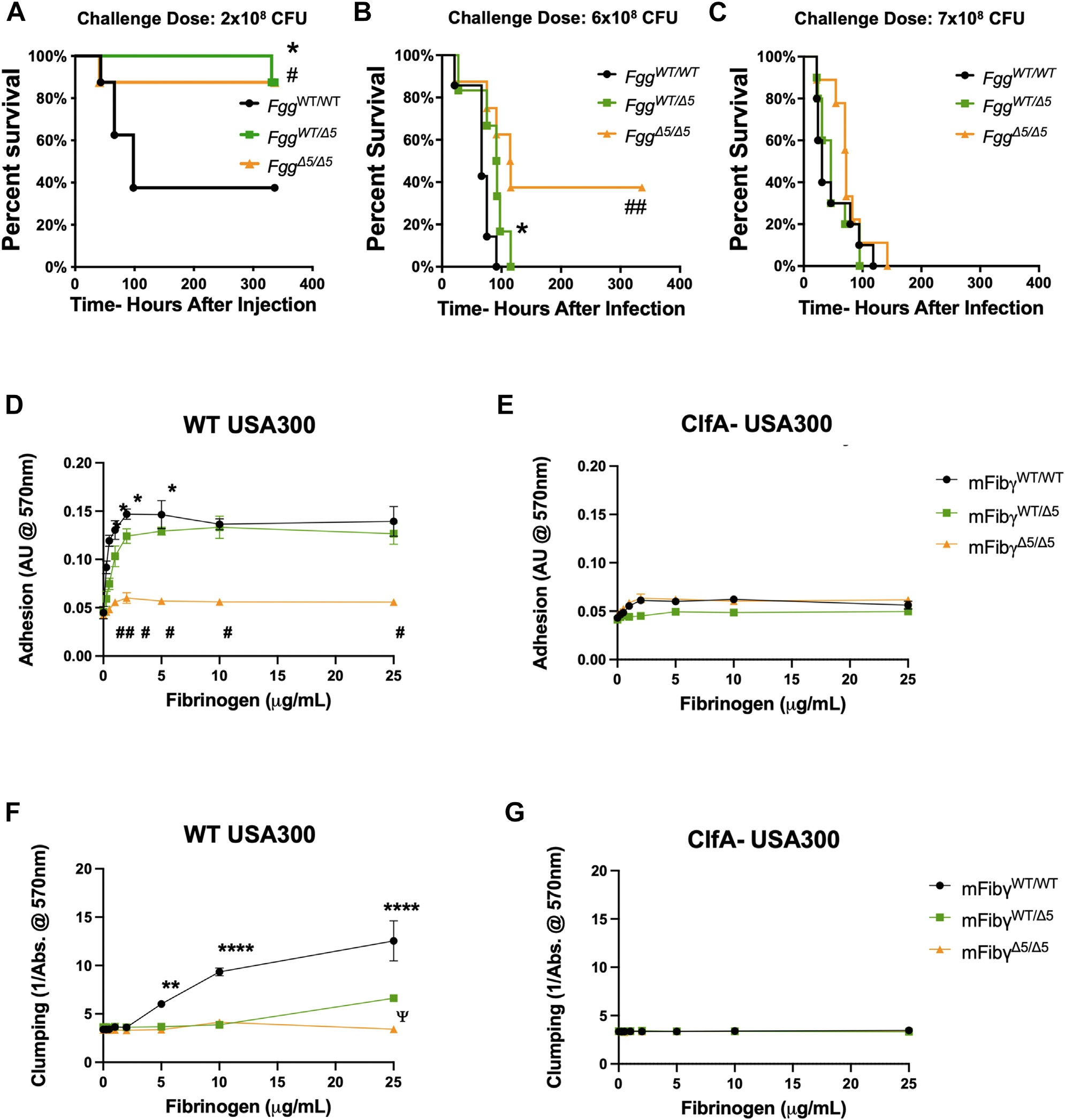
Partial elimination of the ClfA-binding motif results in protection from *S. aureus* septicemia associated with a reduction in fibrinogen-mediated clumping. (A) *Fgg*^*WT/WT*^, *Fgg*^*WT/Δ5*^ and *Fgg*^*Δ5/Δ5*^ mice (*n*=10 per group) were infected via retroorbital injection with 2×10^8^ *S. aureus* USA300, and host survival was monitored over time. Data were analyzed by Kaplan-Meier log-rank analysis with *p < .05 for *Fgg*^*WT/WT*^ vs *Fgg*^*WT/Δ5*^ *and* ^*#*^*p* < .05 for *Fgg*^*WT/WT*^ vs *Fgg*^*Δ5/Δ5*^ mice. (B) *Fgg*^*WT/WT*^, *Fgg*^*WT/Δ5*^, and *Fgg*^*Δ5/Δ5*^ mice (*n*=10 per group) were infected with 6×10^8^ CFUs of *S. aureus* USA300, and host survival was monitored over time. Data were analyzed by Kaplan-Meier log-rank analysis with *p < .05 for *Fgg*^*WT/WT*^ vs *Fgg*^*WT/Δ5*^ *and* ^#^*p* < .05 for *Fgg*^*WT/WT*^ vs *Fgg*^*Δ5/Δ5*^ mice. (B) *Fgg*^*WT/WT*^, *Fgg*^*WT/Δ5*^ , and *Fgg*^*Δ5/Δ5*^ mice (n=10 per group) were infected with 6×10^8^ CFUs of *S. aureus* USA300, and host survival was monitored over time. Data were analyzed by Kaplan-Meier log-rank analysis with *p < .05 for *Fgg*^*WT/WT*^ vs *Fgg*^*WT/Δ5*^ and ^##^*p* < .01 for *Fgg*^*WT/WT*^ vs *Fgg*^*Δ5/Δ5*^. (C) *Fgg*^*WT/WT*^, *Fgg*^*WT/Δ5*^, and *Fgg*^*Δ5/Δ5*^ mice (*n*=10 per group) were infected with 7×108 CFUs of *S. aureus* USA300, and host survival was monitored over time. Data were analyzed by Kaplan-Meier log-rank analysis. Adhesion of (D) WT USA300 or (E) ClfA- USA300 bacteria to immobilized mFibγ^WT/WT^, mFibγ^WT/Δ5^, and mFibγ^Δ5/Δ5^. Data are presented as the mean ± SEM and analyzed by 2-way analysis of variance with Tukey’s multiple comparisons test. ^#^*p* < .0001 for mFibγ^Δ5/Δ5^ vs mFibγ^WT/WT^ and mFibγ^WT/Δ5^; *p < .05 for mFibγ^WT/WT^ vs mFibγ^WT/Δ5^. Clumping of (F) WT USA300 or (G) ClfA- USA300 mediated by mFibγ^WT/WT^, mFibγ^WT/Δ5^, and mFibγ^Δ5/Δ5^. Data are presented as the mean ± SEM and analyzed by 2-way analysis of variance with Tukey’s multiple comparisons test. ^**^p < .01 and ^****^p < .0001 for mFibγ^WT/WT^ vs mFibγ^WT/Δ5^ and mFibγ^Δ5/Δ5^. ^Ψ^*p* < .05 for mFibγ^WT/Δ5^ vs mFibγ^Δ5/Δ5^.

In previous studies, WT mice were shown to rapidly succumb to an intravenous *S. aureus* infection whereas homozygous fibrinogen γ^Δ5^ (ie, *Fgg*^*Δ5/Δ5*^) mice displayed markedly improved host survival. The molecular basis for this observation was linked to elimination of the C-terminal fibrinogen γ ‘AGDV’ binding motif on fibrinogen that was shown to be required for binding to the *S. aureus* virulence factor ClfA [18]. Here, we sought to determine whether a protective benefit would be conferred if only a fraction of the circulating fibrinogen was in the form of fibrinogen γ^Δ5^. *Fgg*^*WT/WT*^, *Fgg*^*WT/Δ5*^, and *Fgg*^*Δ5/Δ5*^ mice were administered an intravenous infection with S. aureus USA300 and monitored. At an infection dose of 2×10^8^ CFUs, both *Fgg*^*WT/Δ5*^ and _*Fgg*_^*Δ5/Δ5*^ mice displayed similar survival phenotypes, showing a significant advantage over infected *Fgg*^*WT/WT*^ mice (Figure 1A). At a higher infection dose of 6×10^8^ CFUs, *Fgg*^*WT/Δ5*^ and *Fgg*^*Δ5/Δ5*^ mice still displayed a significant survival advantage over *Fgg*^*WT/WT*^ mice, although all the *Fgg*^*WT/Δ5*^ mice eventually succumbed to the infection (Figure 1B). At the highest infection dose analyzed of 7×10^8^ CFUs, all mice succumbed to the infection with no genotype-dependent difference in survival times (Figure 1C). These findings indicate that there can be a significant benefit to the host based on infection dose with *S. aureus* sepsis, even if only a portion of the circulating fibrinogen lacks the C-terminal AGDV motif of the γ-chain.

We next evaluated whether the protection in *Fgg*^*WT/Δ5*^ and *Fgg* ^*Δ5/Δ5*^ mice was linked to altered interactions between fibrinogen and the bacteria. Adhesion of WT *S. aureus* USA300 to immobilized fibrinogen was analyzed. A modest but statistically significant reduction was observed in *S. aureus* adhesion to fibrinogen from *Fgg*^*WT/Δ5*^ (mFibγ^WT/Δ5^) relative to that from *Fgg*^*WT/WT*^ (mFibγ^WT/WT^) mice at low plating concentrations of fibrinogen (ie, 0.25 to 5 μg/mL), but no differences were observed between mFibγ^WT/WT^ and mFibγ^WT/Δ5^ at higher plating concentrations (ie, 10 to 25 μg/mL) once binding became saturable (Figure 1D). Little to no adhesion was observed for WT USA300 to fibrinogen from *Fgg*^*Δ5/Δ5*^ (mFibγ^Δ5/Δ5^) (Figure 1D), as shown previously [18,23]. In addition, ClfA- USA300 did not bind to fibrinogen from any of the mouse strains (Figure 1E), similar to previous results [18,23]. Clumping analyses of USA300 in fibrinogen solutions were also analyzed. Here, mFibγ^WT/WT^ supported a dose-dependent increase in clump formation of WT USA300 with no clumping observed in solutions of mFibγ^Δ5/Δ5^ (Figure 1F), similar to previous findings [23]. Notably, clumping was significantly reduced in solutions of mFibγ^WT/Δ5^ relative to mFibγ^WT/WT^ with only the highest concentration of fibrinogen analyzed (ie, 25 μg/mL) displaying a signal above background (ie, that detected for mFibγ^Δ5/Δ5^), but clumping even at this concentration of fibrinogen was significantly less than that observed for mFibγ^WT/WT^ (Figure 1F). ClfA- USA300 did not display clumping with fibrinogen purified from any of the mouse genotypes analyzed (Figure 1G).

### 3.2 | Fibrinogen γ′-γ improves survival following septicemia challenge in mice

**FIGURE 2:**
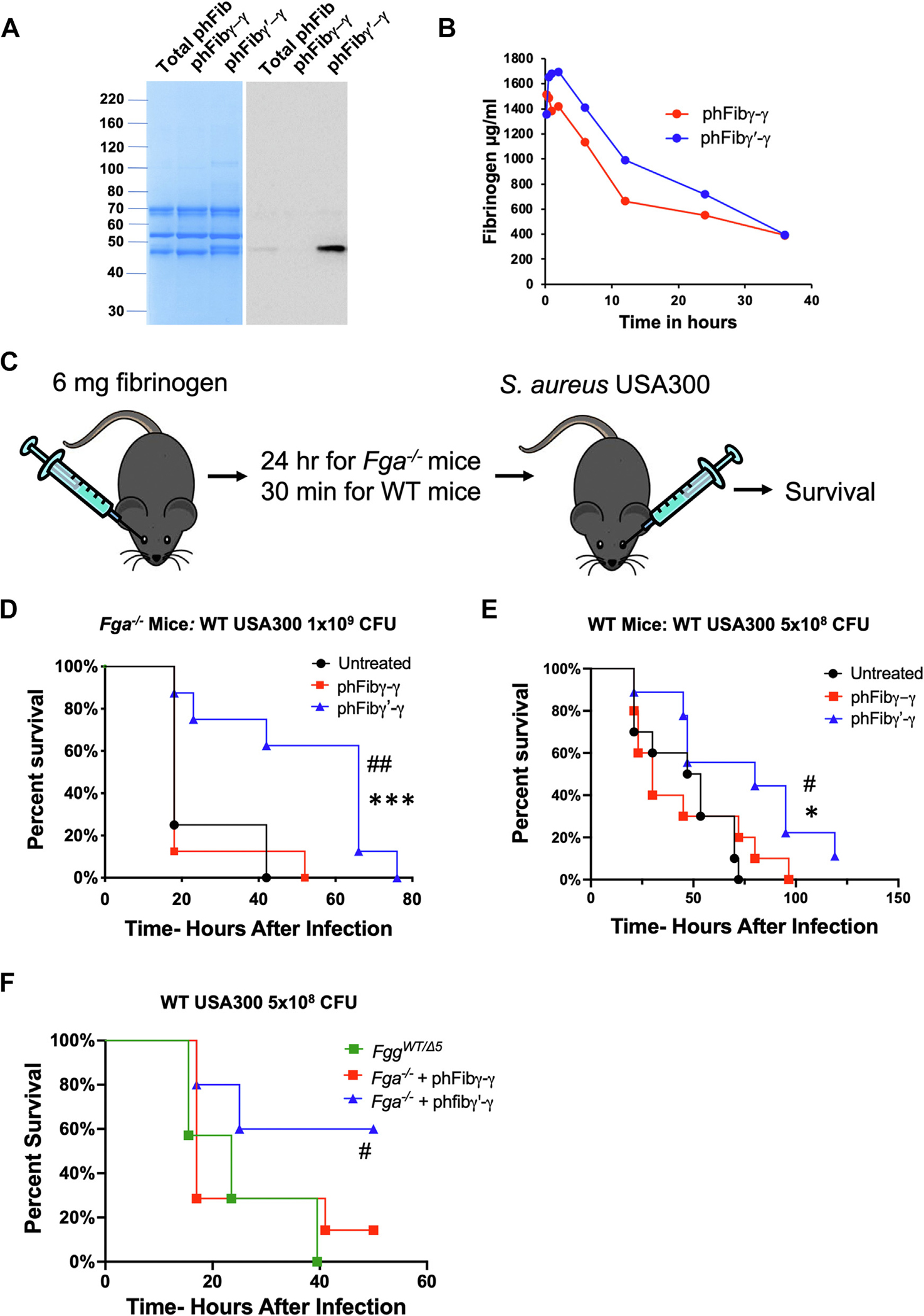
Prophylactic treatment of WT or fibrinogen-deficient mice with fibrinogen γ′ improves survival following septicemia challenge. (A) Purified total phFib, phFibγ-γ, and phFibγ′-γ were analyzed by SDS-PAGE and Coomassie staining (left) and western blot analysis for the fibrinogen γ′ chain (right). (B) ELISA analysis of citrate plasma collected at various time points from C57Bl/6 mice that were injected with 2 mg of phFibγ-γ or phFibγ′-γ (*n*=3 mice per time point for each fibrinogen). (C) Model of prophylactic human fibrinogen treatment followed by intravenous infection with *S. aureus* USA300 in mice. (D) *Fga*^−/−^ mice (*n*=7 per group) were either untreated or injected with 6 mg of phFibγ-γ or phFibγ′-γ followed by retroorbital infection with 1×10^9^ CFUs of *S. aureus* USA300, and survival was monitored over time. Data were analysed by Kaplan-Meier log-rank analysis, with ^##^*p* < .01 for untreated vs phFibγ′-γ and ***p < .001 phFibγ-γ vs phFibγ′-γ. (E) WT mice (*n*=10 per group) were untreated or injected with 6 mg of phFibγ-γ or phFibγ′-γ followed by retroorbital infection with 5×10^8^ CFUs of *S. aureus*, and survival was monitored over time. Data were analyzed by Kaplan-Meier log-rank analysis with ^#^*p* < .05 for untreated vs phFibγ′-γ and *p < .05 for phFibγ-γ vs phFibγ′-γ. (F) *Fgg*^*WT/Δ5*^ and *Fga*^−/−^ mice treated with phFibγ-γ or phFibγ′-γ (n=6–7 mice per group) were injected with 5×10^8^ CFUs of *S. aureus* USA300, and survival was monitored over time. Data were analyzed by Kaplan-Meier log-rank analysis with ^#^*p* < .05 for *Fgg*^*WT/Δ5*^ vs phFibγ′-γ.

Based on the important finding that a 50% reduction in *S. aureus* fibrinogen γ chain binding motifs provided a significant survival benefit at some infection dosages with *S. aureus*, we hypothesized that human fibrinogen γ′-γ would offer the same host protective effects as those observed in *Fgg*^*Δ5*^ mice. To test this hypothesis, we separated total human plasma fibrinogen into purified fibrinogen γ-γ (phFibγ-γ) and fibrinogen γ′-γ (phFibγ′-γ) fractions (Figure 2A). Next, we determined how long phFibγ-γ and phFibγ′-γ would persist in mouse circulation. Each human fibrinogen variant was readily detected in isolated mouse plasma following injection, and both variants displayed a similar half-life of ~15 hours (Figure 2B). Next, a prophylactic approach in which all fibrinogen would be human was tested. Fga^−/−^ mice were administered 6 mg of fibrinogen prior to infection and survival was monitored (Figure 2C). Fga^−/−^ mice receiving only vehicle (ie, untreated) or phFibγ-γ prior to challenge with an aggressive 1×10^9^ CFUs of *S. aureus* USA300 displayed rapid mortality with ~75% of the mice eliminated in less than 24 hours (Figure 2D), and all these animals succumbed to the infection by ~50 hours. In contrast, Fga^−/−^ mice administered phFibγ′-γ displayed a significant survival advantage compared to each of the other groups (Figure 2D). In a second study, WT mice were prophylactically treated with fibrinogen and challenged with dose of 5×10^8^ CFUs of USA300. Even in the presence of normal levels of WT fibrinogen, a similar result was observed in that phFibγ′-γ-treated mice displayed significantly prolonged survival compared with untreated or phFibγ-γ-treated mice (Figure 2E). Finally, an analysis to directly compare *Fgg*^*WT/Δ5*^ and the human fibrinogen variants was performed. Here, *Fga*^−/−^ mice administered phFibγ′-γ displayed a significant survival advantage over the 48-hour observation window relative to both *Fgg*^*WT/Δ5*^ mice and Fga^−/−^ mice carrying phFibγ-γ (Figure 2F). Collectively, these findings suggest that exogenous human fibrinogen γ′ heterodimer can provide significant host protection from *S. aureus* septicemia, even in the context of circulating WT fibrinogen.

### 3.3 | Fibrinogen γ′-γ reduces *S. aureus* organ colonization, protects against reactive changes in circulating blood cells, and suppresses cardiac damage

**FIGURE 3:**
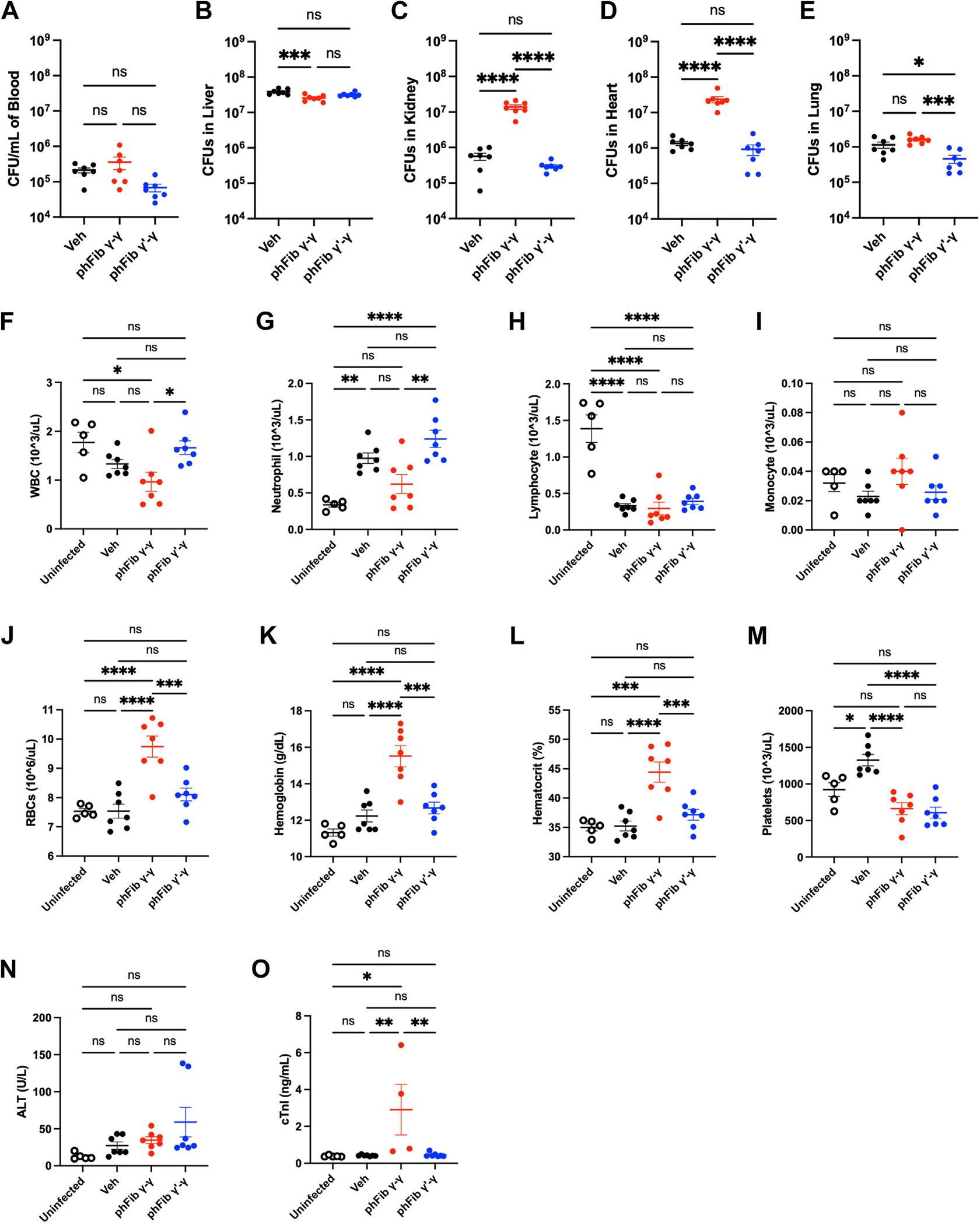
Fga^−/−^ mice reconstituted with fibrinogen γ′-γ and challenged with an intravenous *S. aureus* infection show less organ colonization, protection from reactive changes in complete blood counts, and reduced evidence of end organ damage. *Fga*^−/−^ mice were administered a retroorbital injection of either a vehicle (Veh) control, 6 mg phFibγ-γ, or 6 mg phFibγ′-γ followed by an intravenous infection with 5×10^8^ CFUs of *S. aureus* USA300 24 h later and collection of blood and organ tissues 8 h after infection. Bacterial CFU analyses were performed on (A) blood and tissue homogenates of (B) liver, (C) kidney, (D) heart, and (E) lung. Complete blood counts were performed on uninfected as well as infected mice for analysis of (F) white blood cells (WBCs), (G) neutrophils, (H) lymphocytes, (I) monocytes, (J) red blood cells (RBCs), (K) hemoglobin, (L) hematocrit, and (M) platelets. Plasma was used to analyze circulating tissue damage markers, including (N) alanine aminotransferase (ALT) and (M) cardiac troponin I (cTnI). Data presented as the mean ± SEM and were analyzed by one-way analysis of variance with Tukey_’_s multiple comparisons test. *p < .05, **p < .01, ***p < .001, and ****p < .0001.

To determine the underlying mechanism by which fibrinogen γ′-γ prolongs host survival, *Fga^−/−^* were again treated with either vehicle, phFibγ-γ, or phFibγ′-γ prior to infection with 5×10^8^ CFUs of *S. aureus* USA300. Mice were euthanized 8 hours after infection, a time point immediately preceding the first overt symptoms of the septicemia challenge in control mice. Analysis of bacterial burden in blood revealed no differences regardless of treatment (Figure 3A). Similarly, no fibrinogen treatment-dependent differences were observed in the bacterial burden of liver tissue homogenates (Figure 3B). However, mice treated with phFibγ-γ had significantly higher bacterial burdens than mice administered phFibγ′-γ in tissue homogenates from kidney (Figure 3C), heart (Figure 3D), and lung (Figure 3E). Intriguingly, Fga^−/−^mice administered only vehicle also had significantly reduced numbers of CFUs relative to phFibγ-γ-treated mice, levels nearly identical to phFibγ′-γ-treated mice in these same tissues (Figure 3C–E). These data suggest that WT fibrin(ogen) supports the accumulation and/or proliferation of *S. aureus* in host tissues, whereas eliminating a key virulence factor binding domain on fibrin(ogen) suppresses this activity.

Potential reactive changes in complete blood count were also evaluated. Mice administered phFibγ-γ and challenged with *S. aureus* USA300 showed a significant reduction in white blood cells (WBCs) relative to uninfected mice or mice administered phFibγ′-γ (Figure 3F). The basis for the differences in WBCs was linked to neutrophils and lymphocytes. Whereas all infected animals had higher neutrophil counts relative to uninfected animals, infected mice administered phFibγ-γ had significantly lower neutrophil counts than infected vehicle- or phFibγ′-γ-treated mice (Figure 3G). In addition, lymphocyte counts were significantly higher for uninfected animals relative to all infected groups (Figure 3H). Monocyte counts were not different among all groups (Figure 3I). Intriguingly, *S. aureus*-infected mice administered phFibγ-γ had significantly elevated red blood cells, hemoglobin, and hematocrit (Figure 3J–L) relative to all other groups, including uninfected control mice. Platelet counts were significantly elevated in *S. aureus*-infected mice receiving vehicle control relative to uninfected mice and mice that received either phFibγ-γ or phFibγ′-γ (Figure 3M). The platelet count in mice receiving either fibrinogen was not significantly different from each other or from those in uninfected mice (Figure 3M). There were no differences in the circulating alanine aminotransferase activity among all groups (Figure 3N), while cTnI levels were elevated in phFibγ-γ-treated mice compared with the other groups (Figure 3O), suggesting phFibγ-γ promoted cardiac myocyte damage while phFibγ′-γ was protective against heart injury.

### 3.4 | Fibrinogen γ′-γ supports ClfA-mediated *S. aureus* USA300 adhesion to immobilized fibrinogen and fibrinogen-dependent clumping

**FIGURE 4:**
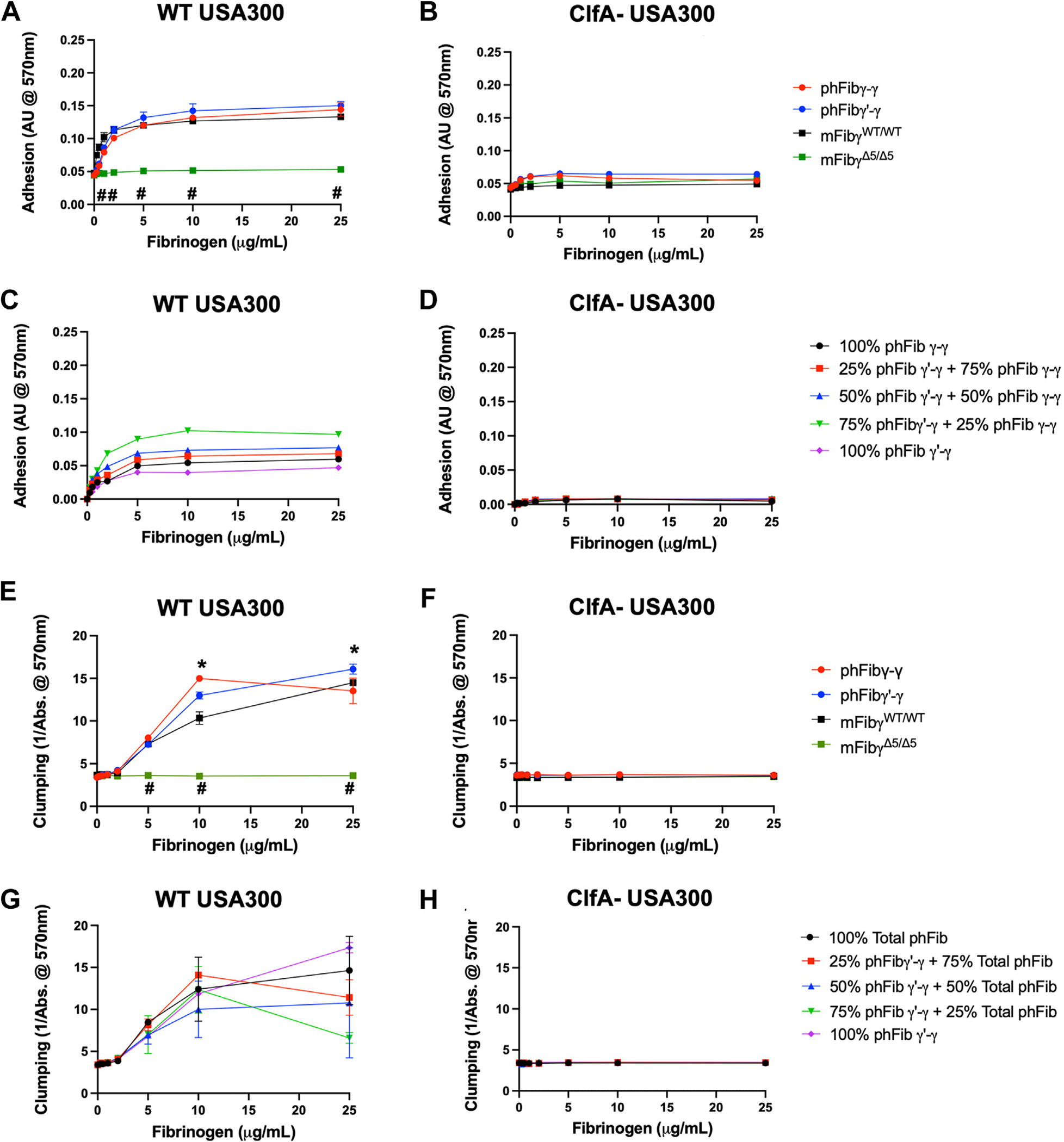
Fibrinogen γ′-γ supports ClfA-mediated adhesion and clumping with *S. aureus* USA300 that is not altered by the presence of total human purified plasma fibrinogen. Adhesion of bacteria to immobilized phFibγ-γ, phFibγ′-γ, mFibγ^WT/WT^, and mFibγ^Δ5/Δ5^ was determined for stationary phase (A) wildtype (WT) *S. aureus* USA300 and (B) ClfA- *S. aureus* USA300. Adhesion of bacteria to immobilized mixes of purified total phFib and phFibγ′-γ was determined (C) WT USA300 *S. aureus* and (D) ClfA- *S. aureus* USA300. Clumping of bacteria in suspension mediated by solutions of phFibγ-γ, phFibγ′-γ, mFibγ^WT/WT^, and mFibγ^Δ5/Δ5^ was determined for (E) WT *S. aureus* USA300 and (F) ClfA- *S. aureus* USA300. Clumping of bacteria in suspension mediated by solutions containing mixtures total phFib and phFibγ′-γ was determined for (E) WT *S. aureus* USA300 and (F) ClfA- *S. aureus* USA300. For each fibrinogen concentration, *n*=3 replicates were performed. Data are expressed as the mean ± SEM and analyzed by 2-way analysis of variance with Tukey^’^s multiple comparison test. ^#^*p* < .001 for mFibγ^WT/WT^ vs mFibγ^Δ5/Δ5^.

We postulated the protection conferred by fibrinogen γ′-γ was linked to an alteration in S*. aureus*–fibrinogen interactions. A previous report indicated that fibrinogen γ′ failed to bind *S. aureus* through ClfA [18]. To confirm and expand on that observation, *in vitro* analyses of bacterial adhesion to immobilized fibrinogen were performed. As shown in Figure 4A, WT *S. aureus* bacteria showed a dose-dependent adhesion at low coating concentrations (ie, <10 μg/mL) that was saturable at higher concentrations (ie, >10 μg/mL) of phFibγ-γ, phFibγ′-γ, and mFibγ^WT/WT^. As a negative control, mFibγ^Δ5/Δ5^ was used, and no adhesion to this fibrinogen mutant was observed with WT *S. aureus* USA300 (Figure 4A). Notably, bacterial adhesion to immobilized fibrinogen was dependent on the *S. aureus* fibrinogen receptor ClfA, as ClfA- *S*. *aureus* showed little, if any, appreciable adhesion to any of the fibrinogen species at any coating concentration (Figure 4B). To determine if an increase in the relative percentage of phFibγ′-γ in the presence of total phFib altered *S. aureus* adhesion, experiments were performed with total purified human fibrinogen (total phFib) and phFibγ′-γ mixes at different ratios ranging from 100% total phFib to 100% phFibγ′-γ. Adhesion of WT *S. aureus* to fibrinogen was detected in all samples (Figure 4C). Although modest differences were detected, no clear distinctions related to the percentage of phFibγ′-γ in the reaction mixture were observed (Figure 4C). The adhesion of *S. aureus* that did occur was dependent on ClfA expression (Figure 4D).

To further characterize the interactions between *S. aureus* and fibrinogen γ′-γ, *in vitro* experiments were performed to determine the ability of fibrinogen in solution to support the formation of *S. aureus* aggregates or ‘clumps.’ Dose-dependent clumping of WT *S. aureus* with phFibγ-γ and mFibγ^WT/WT^ was observed (Figure 4E). No clumping was observed with mFibγ^Δ5/Δ5^ (Figure 4E). Surprisingly, clumping was observed with phFibγ′-γ, indicating that loss of only one of the AGDV motifs on the human fibrinogen molecule is not sufficient to eliminate clumping (Figure 4E). Consistent with previous observations [23], ClfA-*S. aureus* did not support clumping with any of the fibrinogen variants analyzed (Figure 4F). In clumping reactions, altering the relative percentage of phFibγ′-γ did not quantitatively change the overall amount of bacterial clumping with *S. aureus* in the OD assay, but results were variable at the highest concentration of fibrinogen (ie, 25 μg/mL) analyzed (Figure 4G). ClfA- *S. aureus* did not form clumps regardless of the phFibγ′-γ ratio (Figure 4H). Collectively, these data highlight the requirement for ClfA to support clumping and that a human fibrinogen molecule lacking only one AGDV motif still supports clumping.

**FIGURE 5:**
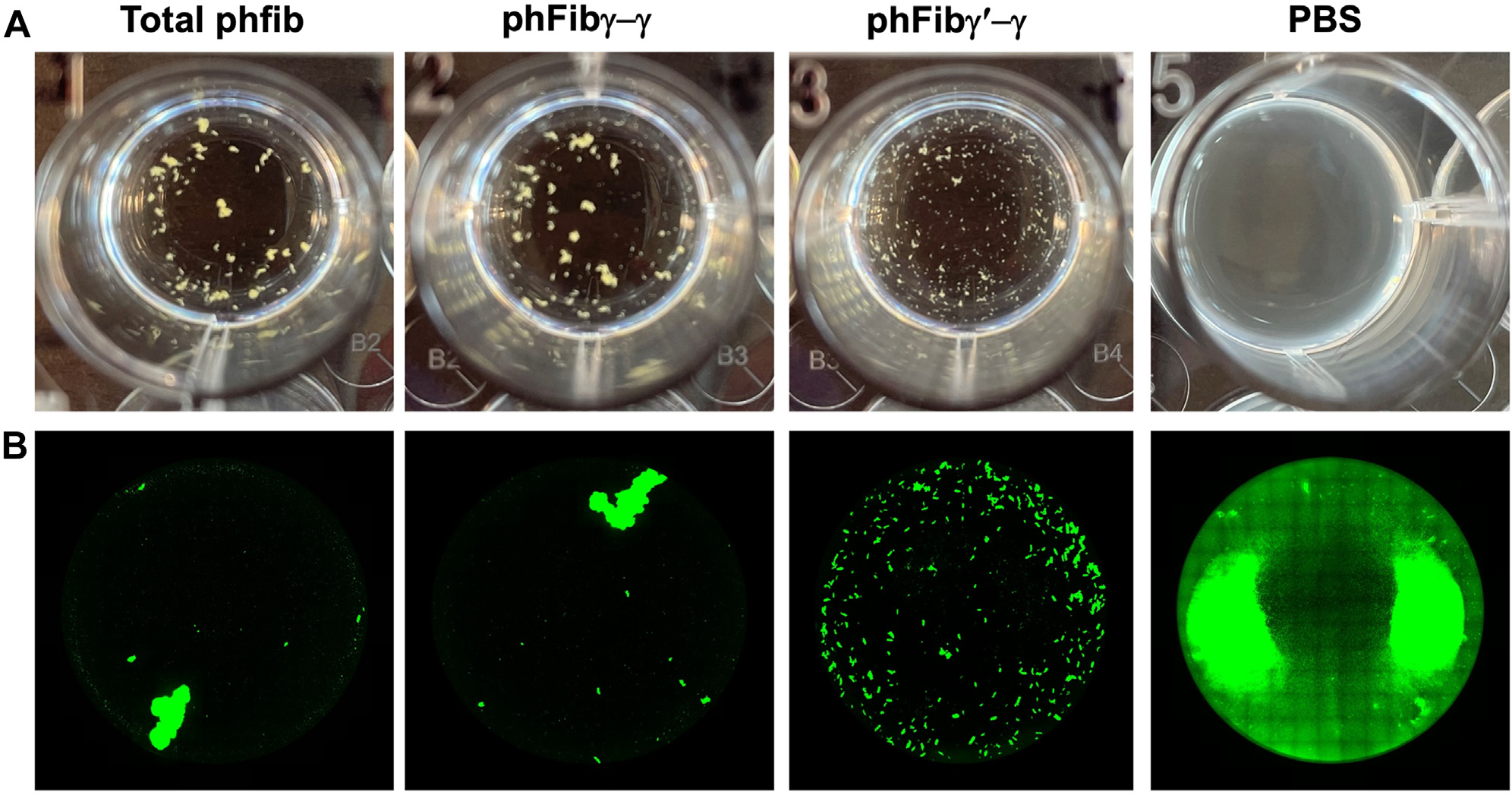
Incubation of *S. aureus* with fibrinogen γ′-γ results in the formation of smaller clumps compared to fibrinogen γ-γ. (A) Clumping reactions of *S. aureus* USA300 in solutions of total phFib, phFibγ-γ, phFibγ′-γ, or phosphate-buffered saline (PBS) vehicle were imaged by brightfield microscopy. (B) Clumping reactions of GFP-expressing *S. aureus* Newman in solutions of total phFib, phFibγ-γ, phFibγ′-γ, or PBS vehicle were imaged by fluorescent microscopy. Note in each case the large clumps generated by total phFib and phFibγ-γ compared to smaller clumps generated by phFibγ′-γ.

The observations that phFibγ′-γ conferred a *S. aureus*-infected host a survival benefit and yet supported quantitatively similar levels of fibrinogen-mediated adhesion and clumping to the microbe suggested there might be qualitative differences in the interactions of *S. aureus* with phFibγ′-γ compared with phFibγ-γ. Given the focus on a blood-stream infection, we postulated that phFibγ′-γ supports the formation of clumps with properties distinct from that observed with phFibγ-γ. Accordingly, fibrinogen-mediated clumps were generated with *S. aureus* USA300 and imaged with standard brightfield microscopy. Here, *S. aureus* clumps generated with phFibγ′-γ appeared smaller and more diffuse overall than clumps generated with total phFib or phFibγ-γ (Figure 5A). Similar studies were performed with GFP-labeled *S. aureus* Newman strain. Here, *S. aureus* clumps formed with phFibγ′-γ again appeared smaller and more diffuse than those formed with total phFib or phFibγ-γ (Figure 5B).

Analyses of plasma-derived phFibγ′-γ′ homodimer are limited by the fact this fibrinogen variant represents <1% of total plasma fibrinogen [20], and thus purification is impractical. To overcome this limitation, we generated sufficient quantities of recombinant fibrinogen γ-γ (rec-hFibγ-γ) and fibrinogen γ′-γ′ (rec-hFibγ′-γ′) for *S. aureus* adhesion and clumping assays (Figure S1A). Here, immobilized rec-hFibγ-γ supported ClfA-mediated adhesion of WT USA300 similar to plasma-purified total hFib, but rec-hFibγ′ -γ′ did not support *S. aureus* adhesion at any coating concentration (Figure S1B–C). Similarly, solutions of rec-hFibγ-γ promoted ClfA-dependent clumping of WT USA300 in suspension similar to total hFib, but no clumping was observed at any fibrinogen concentration evaluated for rec-hFibγ′ -γ′ (Supplementary Figure S1D–E).

### 3.5 | Treatment of mice with fibrinogen γ′-γ extends host survival following infection with *S. aureus* USA300

**FIGURE 6:**
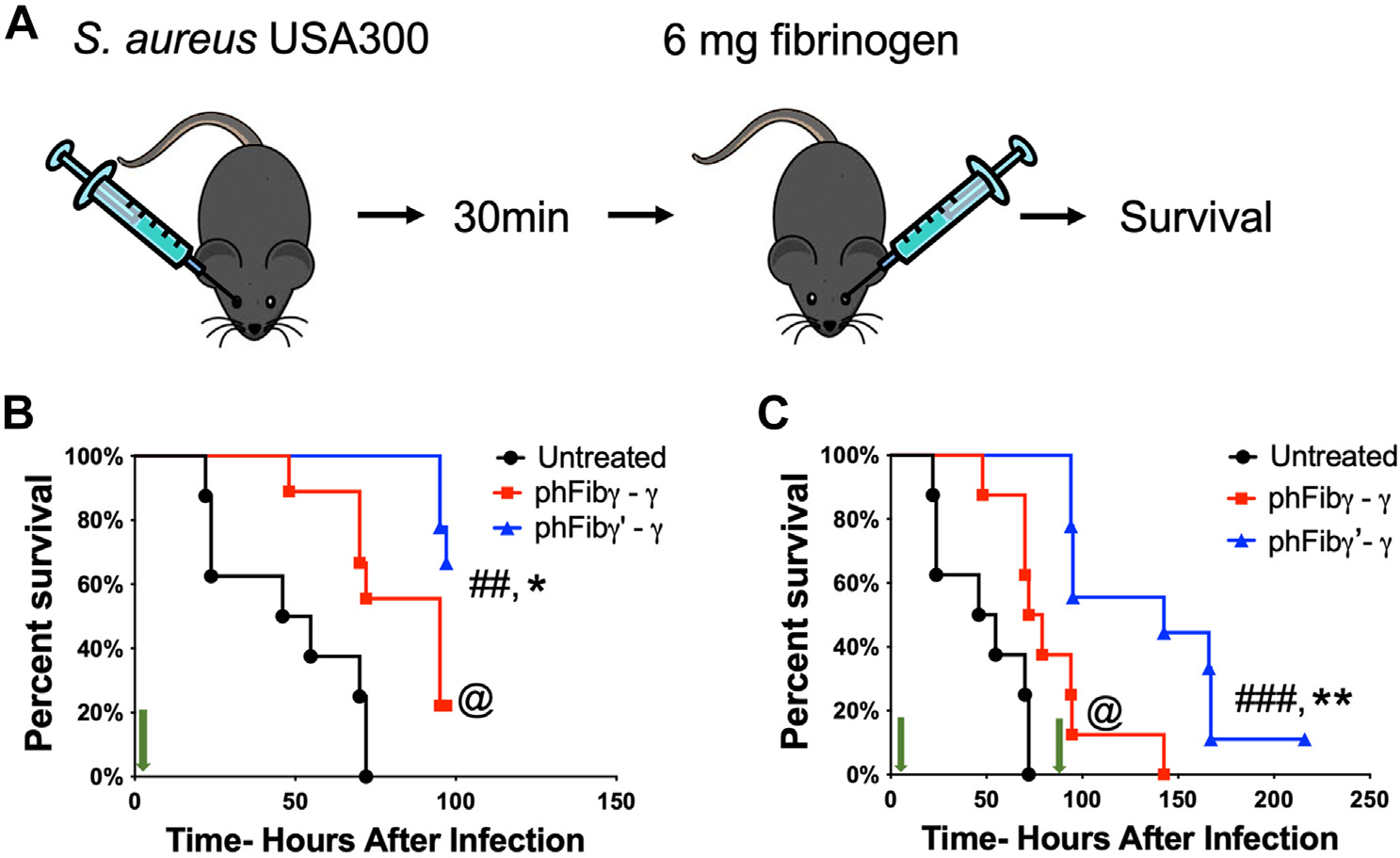
Therapeutic treatment of fibrinogen-deficient mice with fibrinogen g’ improves mouse survival following septicemia. (A) Mouse model of therapeutic fibrinogen treatment after *S. aureus* USA300 infection. *Fga*^−/−^ mice were infected with 5×10^8^ CFUs of *S. aureus* USA300 and were untreated (n=8) or treatment with 6 mg of phFibγ-γ (*n*=8) or phFibγ′-γ (*n*=9) 30 min after the infection. (B) Survival analysis at 90 h after infection and fibrinogen treatment. Data were analyzed by Kaplan-Meier log-rank analysis with ^##^*p* < .01 for untreated vs phFibγ′-γ, ^@^*p* < .01 for untreated vs phFibγ-γ, and *p < .05 phFibγ-γ vs. phFibγ′-γ. (C) After 96 h infection, the remaining mice in the study were treated with a second dose of 6 mg of phFibγ-γ or phFibγ′-γ. Data were analyzed by Kaplan-Meier log-rank analysis with ^###^*p* < .001 for untreated vs phFibγ′-γ, ^@^*p* < .05 for untreated vs phFibγ-γ, and **p < .01 for phFibγ’-γ vs. phFibγ′-γ. For each graph, fibrinogen dosing time is marked with green arrows.

To determine if fibrinogen γ′-γ could provide therapeutic host protection following a systemic *S. aureus* infection, mice were given 6 mg of fibrinogen 30 minutes after *S. aureus* infection (Figure 6A). *Fga*^−/−^ mice were challenged with *S. aureus* USA300 intravenously and left untreated or treated with phFibγ-γ or phFibγ′-γ. Animals treated with phFibγ-γ displayed a significant survival advantage over infected untreated animals, consistent with a recent study showing that elevated fibrinogen levels improve host survival in sepsis [27]. Importantly, mice treated with phFibγ′-γ had a significant survival advantage over the first ~90 hours compared with both untreated and phFibγ-γ-treated mice (Figure 6B). At 96 hours after the infection, surviving mice were redosed with an additional 6 mg of fibrinogen. Following retreatment, mice administered phFibγ′-γ showed prolonged survival relative to the remaining mice treated with phFibγ-γ (Figure 6C). Together, these data suggest that therapeutic fibrinogen γ′ treatment can extend host survival following *S. aureus* septicemia and that continued administration offers extended support to the host.

## 4 | DISCUSSION

In this study, we postulated that a significant increase in host survival following *S. aureus* infection could be appreciated by introducing fibrinogen variants lacking a key *S. aureus* virulence factor binding motif. Building on previous studies, improved survival was observed in mice heterozygous for the γ^Δ5^ mutation in which the binding capacity of fibrinogen to *S. aureus* was reduced. Based on the fibrinogen (AαBβγ)_2_ molecular structure, *Fgg*^*WT/Δ5*^ mice are expected to have a heterogeneous population of fibrinogen molecules with approximately 25% (AαBβγ)_2_, 50% [(AαBβ)_2_γγ^Δ5^], and 25% (AαBβγ^Δ5^)_2_. Thus, we speculated that a fibrinogen heterodimer with half the molecule lacking the final 5 amino acids of the normal γ-chain would be sufficient to confer a benefit to the host. To both test this concept and translate our findings to human fibrinogen variants, we revealed that mice reconstituted with the naturally occurring human fibrinogen γ′-γ displayed significantly improved survival over control animals that was associated with reduced bacterial burden in organ systems, preservation of circulating WBCs/neutrophils, and suppression of tissue damage.

Previous studies documented that fibrinogen γ′-γ can form a fibrin matrix similar to fibrinogen γ-γ [28]. It was also shown that residues critical for binding to several *S. aureus* virulence receptors (eg, ClfA, FnbpA, FnbpB) are absent in fibrinogen γ′ [29–33]. ClfA is of particular interest as this receptor has been shown to directly promote agglutination in blood and thromboembolic lesions in the heart following a bloodstream *S. aureus* infection [9]. In mouse studies, ClfA-deficient *S. aureus* are less virulent following bloodstream infection than WT *S. aureus* [9,18]. A direct link between ClfA, the C-terminal portion of the fibrinogen γ-chain, and the development of *S. aureus* sepsis was also previously documented [18]. Whereas *Fgg*^*Δ5*^ mice show a significant survival advantage over WT mice following infection with WT *S. aureus*, survival of WT and *Fgg*^*Δ5*^ mice is equivalent following infection with ClfA-deficient *S. aureus* [18]. Collectively these previous studies and our current findings suggest that following *S. aureus* bloodstream infection, fibrinogen γ′-γ is sufficient to support hemostasis and maintenance of vascular integrity but has a reduced capacity to bind *S. aureus* bacteria and support virulence.

Our *in vitro* data show that fibrinogen γ′-γ can support *S. aureus* adhesion to immobilized fibrinogen. This observation is not unexpected as fibrinogen γ′-γ encodes one WT γ-chain with a preserved ClfA-binding motif. It was notable that even at fibrinogen coating concentrations as low as 0.25 μg/mL, no significant quantitative difference in binding was observed. This observation may suggest that a fibrinogen molecule coated on a surface is only able to engage bacteria on one half of the fibrinogen (AαBβγ)_2_ molecule and that once binding occurs, bacteria engagement with the other half of the molecule is precluded. It is notable that the binding mechanism between ClfA and fibrinogen is complex and involves residues in the γ-chain beyond the terminal AGDV motif. Recent studies suggest that adhesive function of ClfA for fibrinogen is regulated by mechanical tension as would be experienced under blood flow [34]. This mechanism has been proposed as part of a bridging mechanism between *S. aureus*, fibrinogen, and integrin αVβ_3_ on endothelial cells that contributes to sepsis [35]. In this way, ClfA acts as a type of mechanosensor, but this function could be interrupted with fibrinogen γ′-γ. The bacterial adhesion studies performed here were under static conditions. Conducting similar *S. aureus* binding studies to immobilized fibrinogen under flow would help to resolve whether binding differences to fibrinogen γ′-γ may be appreciated under shear stress conditions.

A net result of the *S. aureus* fibrinogen clumping is to form a ‘shield’ around bacteria protecting the pathogen from host antimicrobial mechanisms and promoting virulence [36–40]. Supporting this concept, *S. aureus* with ClfA genetically eliminated have significantly reduced agglutination in plasma, are less pathogenic, and support the formation of smaller and fewer abscesses in a mouse bacteremia/sepsis model [9,41]. Accordingly, the loss of a ClfA-binding motif and smaller clumps mediated by fibrinogen γ′-γ would be expected to diminish the shielding function of fibrinogen. The reduction in overall bacterial burden in mice with phFibγ′-γ (Figure 3C–E) is consistent with this concept. The mechanism by which fibrinogen γ′-γ potentially perturbs the fibrinogen shield is unknown as it could be a function of an overall reduction in the amount of fibrin(ogen) surrounding *S. aureus* in circulation, an altered fibrin(ogen) shield structure, or both. Moreover, we found that fibrinogen γ′-γ′ is fully deficient in supporting adhesion and clumping of *S. aureus*. With future large-scale production of recombinant fibrinogen, including fibrinogen γ-γ, fibrinogen γ′-γ, and fibrinogen γ′-γ′, it will be possible to determine whether the heterodimer or homodimer confers a greater benefit to the host and the possible mechanisms through which the protective benefit is conferred for each variant.

The C-terminal portion of the fibrinogen γ-chain also mediates interaction with the platelet integrin αIIbβ_3_ receptor that drives fibrinogen-dependent platelet aggregation. Activated platelets express a variety of pattern recognition receptors, phagocytose exogenous antigens, interact with other immune cells (eg, neutrophils), and release numerous soluble mediators (eg, chemokines and cytokines) that can influence the host antimicrobial immune response [42–45]. Previous studies documented that fibrinogen γ′-γ shows an approximate 50% reduction in platelet binding and aggregation [46]. One would speculate that the reduction in fibrinogen–platelet activity conferred by fibrinogen γ′-γ would, if anything, impair the ability of platelets to function as immune mediators to promote antimicrobial activity [47,48]. Additional studies are required to further elucidate the crosstalk between fibrin(ogen), platelets, and *S. aureus* to decipher the potential influence of fibrinogen γ′-γ on infection outcome.

The improved survival observed in fibrinogen γ′-γ treated mice could also be linked to an anticoagulant effect and suppression of thrombin generation or activity. Fibrinogen γ′ can sequester thrombin and thus inhibit its activity, by high-affinity binding of thrombin of exosite 2 to the unique C-terminal γ′ sequence [49–51]. Reconstitution of fibrinogen-deficient plasma with fibrinogen γ′-γ was shown to provide substantially higher thrombin inhibition than reconstitution with normal fibrinogen [52]. Fibrinogen γ′ also exerts anticoagulant effects by diminishing coagulation factors V and VIII activation and increasing the sensitivity to activated protein C [53–55]. Notably, this property is not present in mouse fibrinogen as the similar alternative splicing event of the mouse Fgg gene does not produce a γ′ protein with equivalent properties. Our previous work showed that mice with a mutation resulting in 10% or normal prothrombin levels had a significantly improved survival profile following intravenous infection with *S. aureus* relative to WT mice with normal prothrombin levels [18]. *S. aureus* produces 2 coagulases (ie, *Coa* and *Vwbp*) that can nonproteolytically activate prothrombin and promote fibrin formation [7]. Mice infected with *S. aureus* in which these coagulase proteins were genetically eliminated had a better survival profile relative to WT *S. aureus*-infected mice [9,41]. Notably, mouse fibrinogen γ^Δ5^ does not have the same thrombin modifying activity as human fibrinogen γ′-γ (ie, fibrinogen γ^Δ5^ does not bind and sequester thrombin). Our studies suggest that fibrinogen γ^WT/Δ5^ conferred less host protection than that conferred by fibrinogen γ′-γ. Prophylactic phFibγ′-γ was protective against higher *S. aureus* challenge doses than those observed with *Fgg*^*WT/Δ5*^ (compare, Figure 2D to Figure 1C). Moreover, in a head-to-head comparison, a significant number of fibrinogen-deficient mice supplemented with phFibγ′-γ survived the intravenous *S. aureus* infection, whereas all *Fgg*^*WT/Δ5*^ mice succumbed to infection with the same suspension of bacteria. The findings suggest that for fibrinogen γ^Δ5^ the dominant mechanism of action is loss of binding between the bacteria and fibrinogen, but that phFibγ′-γ confers protection against *S. aureus* infection through multiple pathways. However, formal studies of the various possible mechanisms by which fibrinogen γ′-γ is protective against *S. aureus* infection remain to be performed.

The results presented here highlight the concept that the naturally occurring fibrinogen variant, fibrinogen γ′-γ, confers host protection following a *S. aureus* blood-borne infection. Most notably, we showed that fibrinogen γ′-γ could enhance host survival even when administered to mice already challenged with a blood-borne *S. aureus* infection. Although fibrinogen γ′-γ extended the time of host survival and reduced the overall bacteria burden, it did not promote the complete elimination of the microbes. We postulate that fibrinogen γ′-γ administration may be employed as part of a novel therapeutic strategy for patients with *S. aureus* bacteremia, to extend the therapeutic window of conventional antibiotics and prevent the onset of sepsis in infected patients.

## Supplementary Material

Supplement

## Essentials

- *Staphylococcus aureus* interactions with fibrinogen are a determinant of host defense and pathogen virulence.
- Injection of purified human fibrinogen γ′-γ variant increased survival in a mouse model of *S. aureus* septicemia even in the presence of normal host fibrinogen.
- *S. aureus* adhesion and clumping *in vitro* are not quantitatively reduced with fibrinogen γ′-γ heterodimer, but clumping is qualitatively altered.
- Fibrinogen γ′-γ host protection is associated with reduced *S. aureus* colonization in tissues, lower reactive changes in blood cell counts, and suppression of end organ damage.
