# Supplementary material for: Fibrinogen γ′ promotes host survival during Staphylococcus aureus septicemia in mice
Source: J Thromb Haemost. Author manuscript; Available in PMC 2023 Sep 27. (PMC10528022; doi:10.1016/j.jtha.2023.03.019)
Supplement: Supplement [file NIHMS1909291-supplement-Supplement.docx]

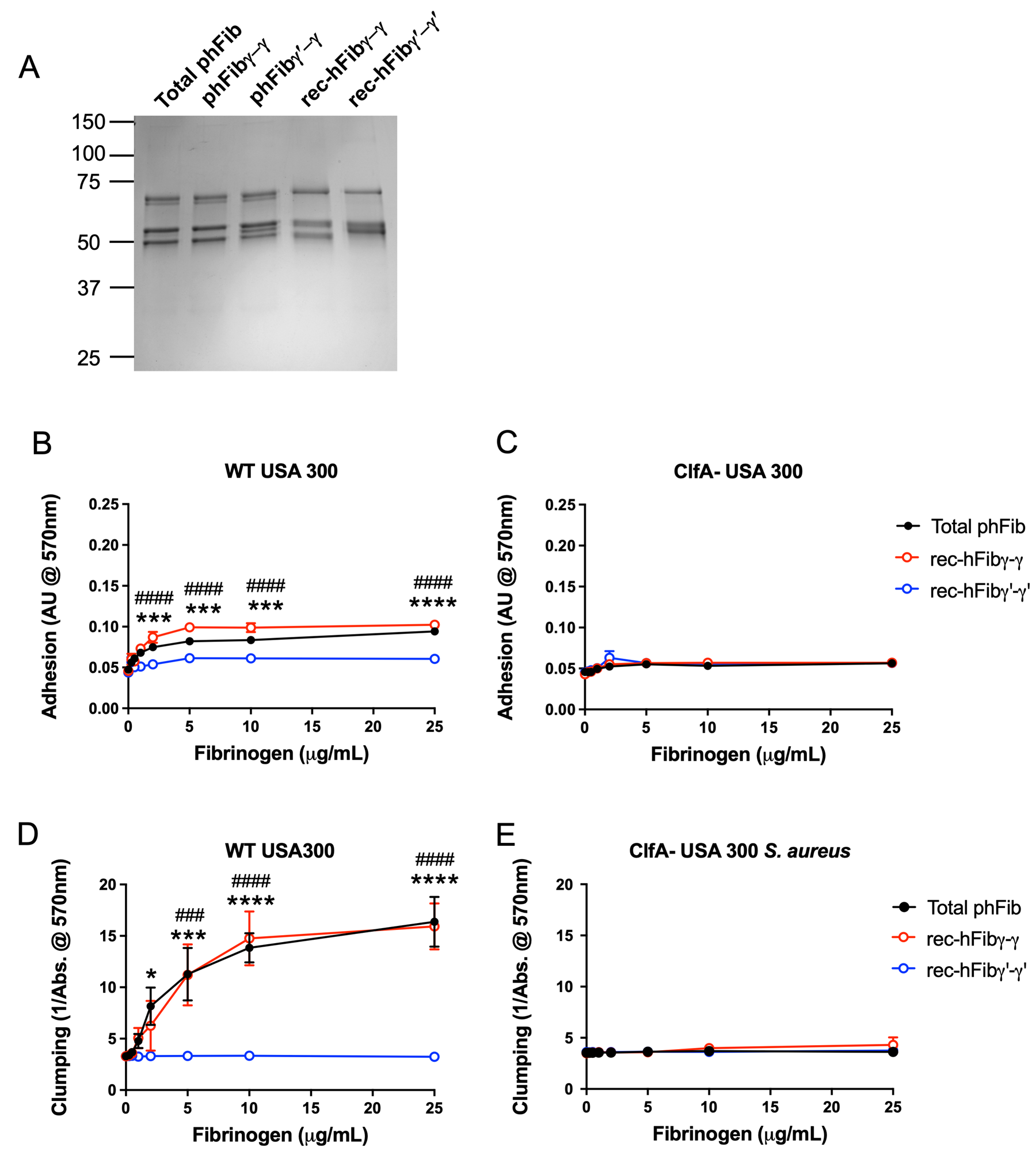


**Figure S1. Recombinant fibrinogen γ'-γ' does not support adhesion and clumping with *S. aureus* USA300.** (A) Purified total phFib, phFibγ-γ, phFibγ'-γ, rec-hFibγ-γ, and rec-hFibγ'-γ' were analyzed by SDS-PAGE and Coomassie staining. Adhesion of bacteria to immobilized Total phFib, rec-hFibγ-γ, and rec-hFibγ'-γ' was determined for stationary phase (B) WT *S. aureus* USA300 and (C) ClfA- *S. aureus* USA300. For each fibrinogen concentration, n=5 replicates were performed. Clumping of bacteria in suspension mediated by solutions of Total phFib, rec-hFibγ-γ, and rec-hFibγ'-γ' was determined for (D) WT *S. aureus* USA300 and (E) ClfA- *S. aureus* USA300. For each fibrinogen concentration, n=4 replicates were performed. Data are expressed as the mean ± SEM and analyzed by 2-way ANOVA with Tukey’s Multiple Comparison Test. **P*<0.05, ****P*<0.001, and *****P*<0.0001 for Total phFib vs. rec-hFib γ'-γ'. ###*P*<0.001 and ####*P*<0.0001 for rec-hFib γ-γ vs rec-hFib γ'-γ'.
